# Mitral Valve Prolapse and Mitral Annular Disjunction Arrhythmic Syndromes: Diagnosis, Risk Stratification and Management

**DOI:** 10.31083/j.rcm2309295

**Published:** 2022-09-05

**Authors:** Panagioula Niarchou, Efstathia Prappa, Ioannis Liatakis, Konstantinos Vlachos, Anastasios Chatziantoniou, Eva Nyktari, Gary Tse, Michael Efremidis, Konstantinos P. Letsas

**Affiliations:** ^1^Second Department of Cardiology, Evangelismos General Hospital of Athens, 10676 Athens, Greece; ^2^Arrhythmia Unit, Onassis Cardiac Surgery Center, 17674 Athens, Greece; ^3^Tianjin Key Laboratory of Ionic-Molecular Function of Cardiovascular Disease, Department of Cardiology, Tianjin Institute of Cardiology, Second Hospital of Tianjin Medical University, 300211 Tianjin, China

**Keywords:** mitral annular disjunction, mitral valve prolapse, ventricular arrhythmias, sudden cardiac death

## Abstract

Although mitral valve prolapse (MVP) is usually considered a benign clinical condition, it has been linked with ventricular arrhythmias and sudden cardiac death in patients with a certain “arrhythmic” phenotype, raising awareness and mandating a specific risk stratification protocol. Mitral annular disjunction (MAD) is considered a “red flag” in malignant MVP syndrome along with bileaflet myxomatous prolapse, female gender, negative or biphasic T waves in the inferior leads, fibrosis in the papillary muscles or inferobasal wall detected by cardiac magnetic resonance imaging and complex arrhythmias of right bundle branch morphology. MAD seems to play a critical role in the chain of morphofunctional abnormalities which lead to increased mechanical stretch and subsequent fibrosis mainly in the papillary muscles, forming the vulnerable anatomic substrate prone to arrhythmogenesis, and associated with long-term severe ventricular arrhythmias. Arrhythmogenesis in MVP/MAD patients is not fully understood but a combination between a substrate and a trigger has been established with premature ventricular contraction triggered ventricular fibrillation being the main mechanism of sudden cardiac death (SCD). Certain characteristics mostly recognized by non-invasive imaging modalities serve as risk factors and can be used to diagnose and identify high risk patients with MAD, while treatment options include catheter ablation, device therapy and surgical intervention. This review focuses on the clinical presentation, the arrhythmogenic substrate, and the incidence of ventricular arrhythmias and SCD in MAD population. The current risk stratification tools in MAD arrhythmogenic entity are discussed.

## 1. Introduction

Mitral annular disjunction (MAD) is an anatomic abnormality which refers to a wide separation between the left atrial wall-mitral valve junction and the basal posterolateral ventricular myocardium that has been recently associated with malignant arrhythmias and sudden cardiac death (SCD) [1, 2, 3, 4, 5]. MAD as a sole entity as well as in the setting of the broader classification of “malignant mitral valve prolapse (MVP) syndrome” are considered potentially arrhythmogenic entitles [6].

In the original description from Hutchins *et al*. [1] in the 1980s in 900 hearts with “floppy” mitral valves, MAD was defined as a wide separation ≥5 mm between the posterior leaflet insertion into the left atrial wall and the LV attachment was found in 92%, suggesting a correlation between this anatomic variant and myxomatous degeneration of the mitral valve. MAD has been reported to exist in 6% of normal hearts, as a normal anatomic variant [1]. The first echocardiographic description comes from Erikson *et al*. [2] in 2005 where MAD was observed by transesophageal echocardiography in 99% of patients, referred for surgical repair for advanced mitral valve (MV) disease. To that point, MAD was only studied as an anatomic variant in the context of MVP, possibly accelerating the degenerative process of the MV [1]. Hypermobility and systolic elongation of the posterior mitral annulus as a consequence of MAD was thought to increase mechanical stretch on the valvular apparatus and subsequently lead to myxomatous degeneration [1]. During last decades MAD has been associated with malignant ventricular arrhythmias in the setting of MVP [3, 5]. However, recent evidence supports its arrhythmogenic entity even in subjects without MVP [6].

Thus, MAD is now considered a “red flag” in malignant MVP syndrome along with bileaflet myxomatous prolapse, female gender, negative or biphasic T waves in the inferior leads, fibrosis in the papillary muscles or inferobasal wall detected by cardiac magnetic resonance (CMR) and complex arrhythmias of right bundle branch morphology, while its role in arrhythmogenesis remains to be clarified [3, 6, 7, 8]. This review focuses on the clinical presentation, the arrhythmogenic substrate, and the incidence of ventricular arrhythmias and SCD in MAD population. The current risk stratification tools in this new arrhythmogenic entity are discussed.

## 2. Definition and Diagnosis

**Fig. 1.**
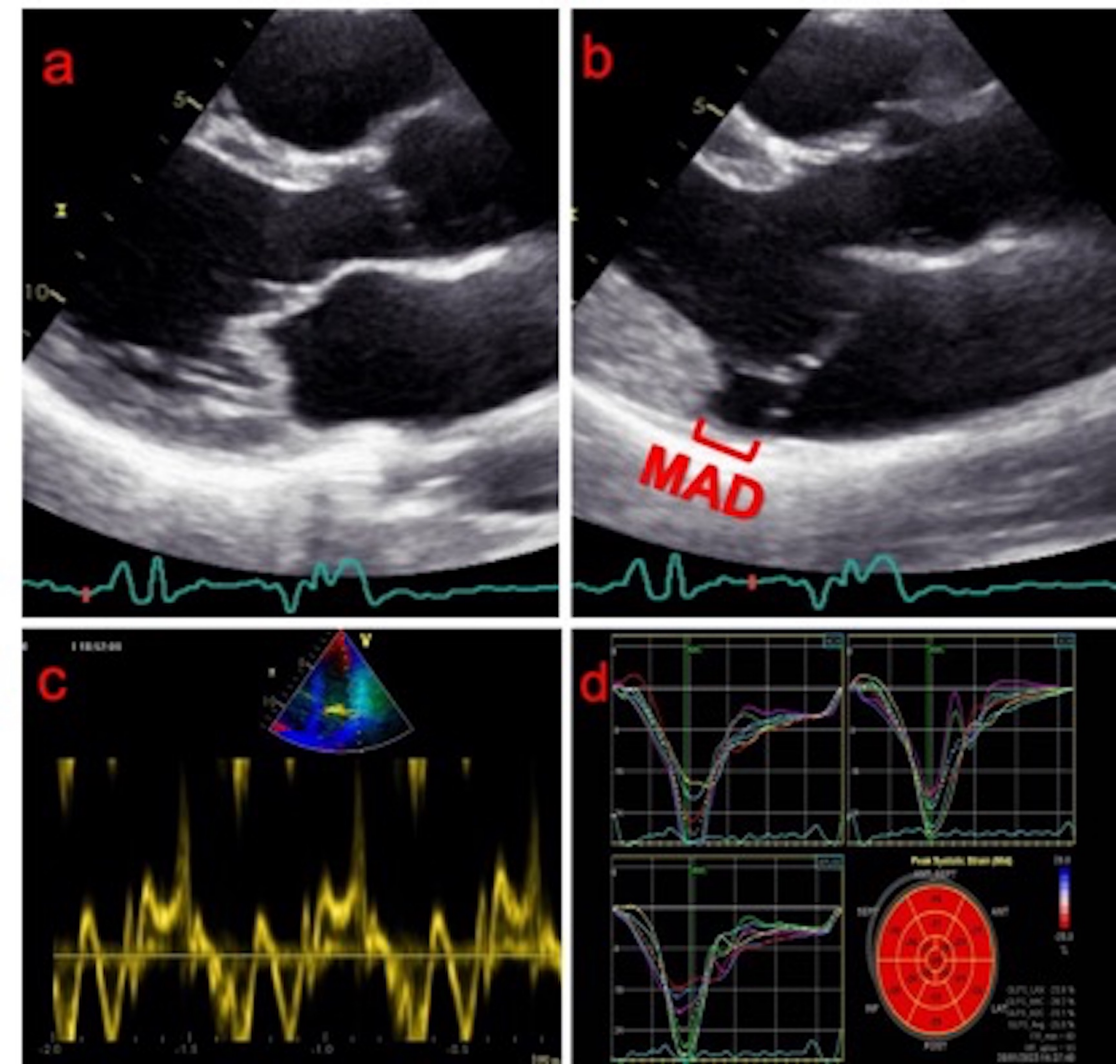
**Echocardiographic findings of mitral valve prolapse and mitral annular disjunction**. (a) Transthoracic parasternal long axis echocardiographic view of a bileaflet mitral valve prolapse during end-diastole. (b) Mitral annular disjunction is revealed during end-systole. (c) The “Pickelhaube” sign (spiked systolic lateral mitral annular velocity >16 cm/s) may serve as an early indicator of mechanical stress even in the absence of fibrosis. (d) Longitudinal strain (GLS) with supranormal values in the basic inferior/lateral wall of the left ventricle.

MVP affects 1–3% of the general population [9] and is classically defined as a superior displacement of MV leaflets above the mitral annulus >2 mm, measured echocardiographically in the parasternal long axis view during systole [10]. There are two phenotypes described. The first one refers to the classic form of Barlow’s disease which is characterized by myxomatous degeneration of the MV, with bileaflet prolapse of redundant and thickened leaflets (≥5 mm) (Fig. 1a), while the second one is characterized by fibroelastic deficiency (FED) resulting in thinner leaflets with segmental prolapse due to connective tissue abnormalities [11, 12]. There are also familial and genetic patterns described, with filamin C (FLNC) gene mutations being related to arrhythmogenic forms of MVP.

**Fig. 2.**
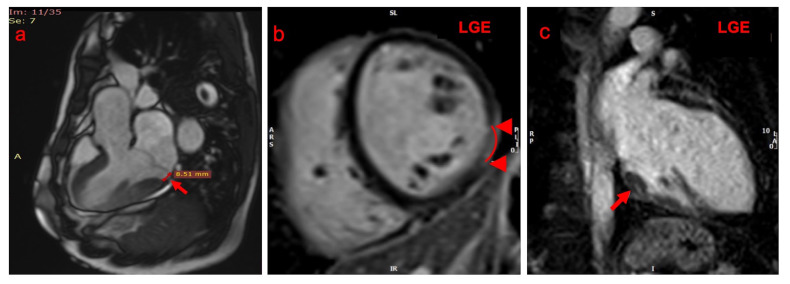
**Cardiac magnetic resonance findings of mitral annular disjunction (MAD) arrhythmic syndrome**. (a) MAD with a distance of 8.51 mm in the posterolateral wall (red arrow). (b,c) Late gadolinium enhancement (LGE) localized at the posteromedial papillary muscle and at the inferior wall of the left ventricle (red arrows).

In pathological studies, MAD has been mainly related to MVP with floppy mitral valve and myxomatous degeneration rather than FED [1, 13, 14]. MAD can be easily detected by transthoracic echocardiography (TTE) [2], and measured in the parasternal long axis view during end-systole (Fig. 1b). It mainly affects the area under the posterior mitral leaflet, which is more prone to mechanical stretch as opposed to the more rigid aorto-mitral continuity [14]. A threshold ≥5 mm was first adopted [1], but a cutoff of 2 mm has been also proposed and used in some studies [14]. Longitudinal MAD distance and circumferential extension can be accurately identified by CMR [15] with reports of MAD area ranging between 30°–240° (median 150°), corresponding to merely 2/3 of the annular circumference [6] (Fig. 2a). Cardiac computed tomography (CT) is also used in certain studies to characterize MAD [16].

A clear description of the patho-morphological substrate of MAD is still lacking. In a recent study, Faletra *et al*. [17] evaluated MVP patients with an echocardiographic diagnosis of MAD by CMR, and based on the hinge line of posterior leaflet in the diastolic phase concluded to two possible anatomic phenotypes: “pseudo”-MAD and true MAD. The first refers to the juxtaposition of posterior leaflet on the atrial wall in systole which imitates MAD, although the insertion point of the leaflet is normal, while the second one refers to true displacement of the hinge point into the atrial wall, also observed in diastole. This true MAD area is formed from a subannular membrane [18] and it is not clarified whether MVP patients share the one or the other phenotype [17].

In recent studies, MAD was identified in approximately 16–55% of MVP patients, with varying results but lower prevalence than previously reported, maybe due to different imaging modalities used and populations studied [19, 20]. In a series of 185 patients, Konda *et al*. [14] reported that MAD is detected in 24% of patients with severe mitral regurgitation and in 90% of patients with Barlow’s syndrome, while in a recently published review where 19 studies where included, MAD prevalence was reported to be 50.8% in patients with myxomatous mitral valve, 32.6% in patients with MVP and 25.9% in patients with a floppy mitral valve and severe mitral regurgitation [21]. Of note, in a recent retrospective study where cardiac CTs of structurally normal hearts were 3D analyzed, MAD was seen in 96% of patients as a normal “phenomenon” of the mitral annulus formation [16].

## 3. SCD Prevalence

The prevalence of SCD in MVP population is low and estimated to be between 16 to 41 per 10000 per year (0.2% to 0.4%) [22]. Yet the real prevalence is difficult to be determined. Among patients with MVP, only a few will suffer SCD, however given the prevalence of MVP in the general population [23], the total number of patients at risk is significant. In Italian autopsy series, as reported by Basso *et al*. [22], MVP was identified as the cause of death in 7% of young adults <40 years, and in 13% amongst females, representing the first fatal cause in this second group. Autopsy studies often report MVP as a cardiac finding in an otherwise structurally normal heart, but due to the uncertain cause-effect relationship between MVP finding at autopsy and SCD, it is not classified as a fatal structural abnormality [24]. This observation is further supported by a meta-analysis of autopsy studies where 22% of deaths were reported unexplained and 11.7% of them exhibited MVP [23]. Yet, the arrhythmic prevalence of MAD itself remains to be determined taking into consideration that in the recent 10-year cohort of MAD patients, this clinical entity was not linked to excess mortality, but it was significantly correlated with severe ventricular arrhythmias in long-term follow-up [25].

## 4. Ventricular Arrhythmias in MAD: Origin, Triggers and Anatomic Substrate

Arrhythmogenesis in MVP/MAD patients is not fully understood but a combination between a substrate and a trigger has been established with PVC triggered VF being the main mechanism of SCD [26]. There is a wide spectrum of ventricular arrhythmias in MVP-MAD population ranging from premature ventricular complexes (PVCs) to sustained ventricular arrhythmias [ventricular tachycardia/ventricular fibrillation (VT/VF)] [27]. In the recently announced EHRA consensus statement on arrhythmic MVP/MAD complex, “arrhythmogenic MVP” was defined as the presence of MVP, with or without MAD, in patients with frequent (≥5% of total PVC burden) or complex arrhythmias (NSVT/VT/VF) in the absence of any other well-defined arrhythmic substrate [28]. On the other hand, it has been recently reported that MAD arrhythmic syndrome can be the manifestation of a concealed proarrhythmic genetic substrate which underlies, and mechanical stretch can serve as the trigger for arrhythmias on this vulnerable-in the presence of fibrosis-substrate [29]. The incidence of PVCs in MVP population has been reported to be between 49 and 85% [26], while in a recent cohort of 595 MVP patients ventricular arrhythmias occurred in 43% [30]. Localized reentry, triggered activity and autonomic system abnormalities has been proposed to be implicated in arrhythmogenesis of MVP/MAD population [26, 27, 31].

Until now, malignant arrhythmias and SCD in MVP patients has been linked to severe mitral regurgitation and subsequent left ventricular remodeling [26]. However, in out of hospital cardiac arrest (OHCA) survivors with bileaflet MVP, Sriram *et al*. [7] have demonstrated an association between MVP phenotype and arrhythmic SCD even in the absence of significant mitral regurgitation. In addition, in a study of Essayagh *et al*. [30] which included 595 MVP patients, severe ventricular arrhythmia was not associated with mitral valve regurgitation severity.

**Fig. 3.**
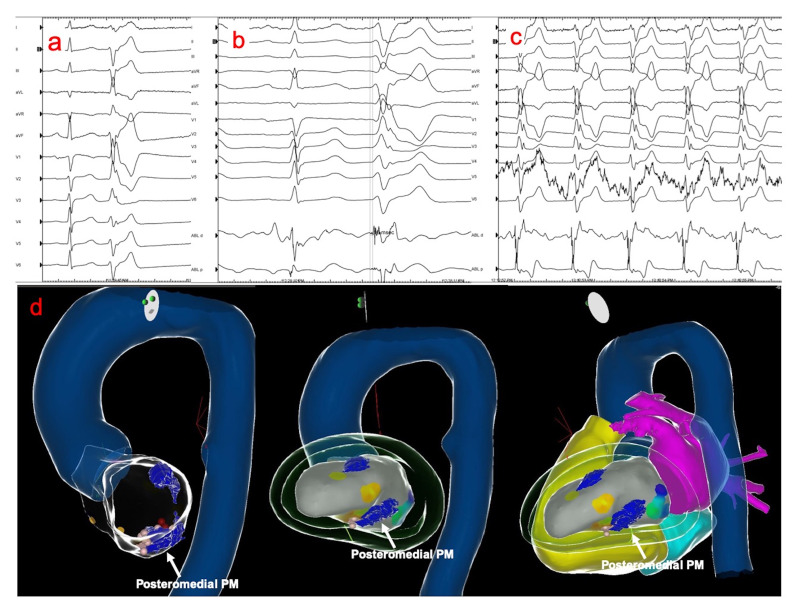
**Electroanatomical mapping and ablation of premature ventricular contraction (PVC) arising from the posteromedial papillary muscle**. (a) Typical PVC arising from the posteromedial papillary muscle (left superior axis with RBBB morphology). (b) PVC mapping demonstrating Purkinje potentials preceding the local ventricular activation. (c) Catheter ablation at the earliest activation site led to “warm-up” effect with the same PVC morphology. (d) Merge of electroanatomical 3-D and cardiac CT models demonstrating the papillary muscles (marked in blue). The posteromedial papillary muscle along with the ablation points are shown in multiple views (white arrows).

The origin of arrhythmias mainly involves the papillary muscles (PM) and the outflow tract in areas near the MV apparatus [7, 32, 33]. Among survivors of OHCA with documented VT/VF, patients with bileaflet MVP had higher burden of PVCs and complex ventricular arrhythmias originating from the outflow tract and PM or fascicles, in an alternating fashion [7, 8]. The close proximity of the arrhythmogenic ectopic activity to the MV apparatus suggests an association between an anatomic substrate (mechanical stretch of the PM due to hypermobile mitral annulus and consequent fibrosis) and arrhythmogenesis [26, 33]. The development of fibrosis in the left ventricular myocardium close to the MV apparatus (100% patchy replacement-type fibrosis at the level of PM and 80% sub-endocardial and mid-mural fibrosis at the infero-basal wall) has been associated with ventricular arrhythmias of Right Bundle Branch Block (RBBB) morphology and SCD in MVP cases [3]. More precisely, the posterior PM seems to be the main origin of arrhythmias in MVP patients (Fig. 3a) and has been correlated with late gadolinium enhancement (LGE) in CMR [32].

MAD seems to play a central role in the structural substrate predisposing to malignant arrhythmias [26]. First, Carmo *et al*. [5] in 2010 demonstrated that MAD, is associated with frequent PVCs and non-sustained ventricular tachycardia (NSVT) in a population of MVP patients in comparison to those with MVP but without MAD. More precisely they correlated MAD length >8.5 mm with the occurrence of NSVT, suggesting a critical role of this variant in the chain of morphofunctional abnormalities of the mitral valve annulus which form the suitable anatomic substrate for malignant ventricular arrhythmias. Deijgaard *et al*. [6] went a step further by describing the MAD arrhythmic syndrome in 116 patients with MAD, where 12% of them suffered severe arrhythmic events that were not linked to MVP, thus revealing MAD itself as an arrhythmogenic entity. The chain of morphofunctional annular abnormalities in MVP patients with MAD led to Padhua hypothesis [26]. When MAD is present, functionally the mitral valve annulus is decoupled from the left ventricular myocardium [34]. This disruption of the ventriculo-mitral unit leads to hypermobility and paradoxical systolic motion of the annulus [34]. Normally the annulus follows contraction of the left ventricle during cardiac cycle [35]. In MAD, the annulus moves according to left atrium and instead of deepening its saddle shape in systole, it expands and flattens forcing the left ventricular basal myocardium to move outwards in systole and inwards in diastole [34, 36]. The flattening of the mitral annulus increases mechanical stretch on the mitral leaflets [37] and PM causing PM elongation and resulting in leaflet degeneration, regional hypertrophy and subsequent fibrosis [4, 38]. This hypothesis is further supported by CMR findings where LGE enhancement is confirmed in the areas prone to mechanical stretch [3, 15] (Fig. 2). Enhanced basal left ventricular systolic deformation (supranormal in inferolateral segments) along with high tissue velocities in the basal lateral wall, regional hypertrophy and left ventricular dilatation both in CMR and echocardiographic studies of MVP and MAD population suggest that MAD is a disease not only of the MV but also of the adjacent myocardium [15, 39, 40]. There are reports that fibrosis can present later in the course of the disease and arrhythmias have been documented even in the absence of LGE in CMR [7, 33] in few cardiac arrest survivors. This finding underlines mechanical stretch as the main arrhythmic trigger.

A traumatized fibrotic myocardium and/or a diseased Purkinje tissue are mutually involved in arrythmogenesis [26], the first acting as the vulnerable substrate and the latter as the trigger by means of afterdepolarizations and abnormal automaticity [7, 31, 41]. PVCs arising from Purkinje fibers were identified as VF trigger in the electrophysiological study (EPS) in 6 out of 6 cardiac arrest survivors and as the dominant ventricular ectopy site in 5 out of 8 patients with complex arrhythmias, all with bileaflet MVP [33]. Previous publications suggest that papillary muscles represent an anatomic structure potentially triggering VF even in the normal heart [42]. More specifically, in idiopathic VF populations short coupled ventricular extrasystoles from PMs were preceded by Purkinje-like potentials (PLPs) and were the main mechanism initiating VF [32, 43]. The Purkinje fibers are the terminal part of the ventricular conducting system lying at the subendocardium and extending from the interventricular septum to PMs and the lateral ventricular walls [44]. They are characterized by fast conducting properties and automaticity while their complex architecture favors re-entrant circuits and maintenance of ventricular arrhythmias and VF [41, 45]. In cardiac arrest survivors with documented VF and MVP, PLPs were recorded in the PMs [33]. In the series of Syed *et al*. [33], the presence of diseased fascicular/Purkinje system was confirmed by the presence of fractionated and abnormal split potentials in asymptomatic MVP patients with inducible VF in the standard EPS, underlying the Purkinje tissue disease as the main electrophysiologic abnormality implicated in arrhythmogenesis. Mechanical stretch to the PMs from hypermobile mitral annulus in patients with MAD may alter the electrophysiologic properties of surrounding tissue shortening the action potential duration and prolonging the ventricular refractory period [7, 44], thus facilitating re-entry in areas of fibrotic myocardium and triggered activity by stretch-activated afterdepolarizations in the local Purkinje tissue [43]. This case is supported by EPS findings which revealed areas of slow conduction in close proximity with MV [33]. Autonomic nervous system dysfunction seems to contribute in arrhythmogenesis with most of PM ventricular arrhythmias being catecholamine sensitive [46]. High sympathetic tone predisposes to increased ventricular ectopy, while enhanced catecholamine levels lead to alterations in Ca+ levels in sarcoplasmatic reticulum [7]. As a result, delayed afterdepolarizations and ventricular ectopy emerge [41].

## 5. Risk Stratification

Risk stratification in this population is highly demanding. The incidence of SCD is relatively low, but over-representation of MVP in unexplained SCD cohorts along with the high prevalence of MVP in the general population indicates a large population at risk [20], which remains to be identified. There are several risk factors reported including ECG abnormalities, morphofunctional characteristics, echocardiographic and CMR findings [7, 8, 26]. The emerging role of EPS is yet to be clarified and added to a risk stratification model aiming to identify the “high risk“ patient.

SCD seems to be of great importance in the young and female patients with MVP [7, 8]. Conflicting evidence concerning the prevalence of SCD according to age exist [8, 25]. Studies in cardiac arrest survivors established a certain high risk clinical profile in young women with syncope, bileaflet myxomatous MVP, MAD, mild or mild-to-moderate mitral regurgitation, biphasic or inverted T waves in the inferior leads and frequent PVCs originating from outflow tract or papillary muscle [7, 8].

In unpublished data from our group, fibrosis detected by CMR, PVCs morphology (RBBB with left superior axis) and positive programmed ventricular stimulation during EPS seem to be the high risk features. In the largest cohort of MAD-MVP patients published in 2021, Essayagh *et al*. [25] concluded that the presence of MAD is independently associated with long term excess incidence of clinical arrhythmic events proving the progressive course of the substrate and mandating careful monitoring for malignant arrhythmias in the long term. These findings that are suggestive of a “progressive disease” are in line with a catheter ablation study in MVP patients where 5 out of 15 patients with successful ablation of dominant PVC foci presented during follow up with hemodynamically significant VT/VF arising from a different site [47]. Of note, all of these patients had inducible VF in prior EPS [47].

MAD seems to correlate with regional fibrosis as represented by LGE enhancement in the adjacent myocardium [15, 48, 49] and PMs [46] and these patients are more prone to arrhythmic events as stated by pathologic and EPS studies [3, 6]. Myocardial extracellular volume (ECVsyn) also plays a role in revealing interstitial myocardial fibrosis in MAD-MVP patients [48]. Increased ECVsyn of the basal LV segments was found to have a strong association with MAD and OHCA even in the absence of LGE [48]. Thus CMR with pre- and post-T1 mapping and ECVsyn calculation should be a basic tool in risk stratification of this population [15, 48, 49].

The origin of ventricular ectopic activity strongly correlates with malignant arrhythmia burden and SCD [7, 33]. The prevalence of PVCs in the MVP population is high but complex VTs are more common among SCD population [7]. As previously discussed ventricular ectopy seems to be mainly of LV origin since all SCD victims displayed RBBB morphology ventricular arrhythmias [3]. More robust investigation revealed as the dominant PVC morphology in MVP patients with complex arrhythmias, the one originating from posterior PM [32]. Thus PVCs originating from this area should be considered as a “red flag” and added to risk stratification algorithm [32].

Biphasic and inverted T waves in inferior leads are considered a high risk marker possibly reflecting repolarization abnormalities due to altered contractility in the basal LV segments adjacent to MAD [6, 7, 26]. There are reports of longer corrected QT intervals among arrhythmia patients with MVP but this is not a constant finding with reports varying from 9% to 26% [26, 50]. QT dispersion is also reported to be higher in these patients but both these parameters cannot be strongly associated with arrhythmic risk [51].

Longitudinal MAD distance both in echo [5, 6] and CMR along with systolic curling motion of the posterior mitral annulus [52] correlate with increased risk of ventricular arrhythmia. Precisely, a disjunction length >8.5 mm was associated with NSVT on holter monitoring in a cohort of 38 MVP patients [5] while circumferential area and greater longitudinal MAD distance measured in CMR was an independent risk factor for arrhythmias [6]. Novel echocardiographic findings have also been reported in the MAD-MVP population [13, 53, 54]. Higher annular tissue velocities in the basal lateral segment depicted in the “Pickelhaube” sign [39] as a spiked configuration with velocities >16 cm/s reflect the mechanical stretch on these myocardial regions and can serve as an early indicator of mechanical stress even in the absence of fibrosis (Fig. 1c). Heterogeneity of longitudinal strain has also been reported with supranormal values in basal inferolateral regions [53, 55] reflecting hypercontractility along with higher myocardial work in the same regions [56] (Fig. 1d). Basal to mid LV wall thickness ratio >1.5 were also higher in MVP patients with late gadolinium enhancement than in those without [4]. These findings can be explained by constant stretch leading to increased oxygen demand and oxidative stress but remains to be further confirmed. Mechanical dispersion, already associated with ventricular arrhythmias in patients with cardiac diseases [57], was also found to be higher in arrhythmogenic MVP patients [58].

The role of EPS in MVP population has not been clearly established and remains controversial since the induction of VF is considered a non-specific finding in patients with complex ventricular arrhythmias. Studies in MVP cardiac arrest survivors and ablation population revealed hemodynamically significant VT/VF even in the absence of myocardial scar [8, 33]. These patients had a high burden of PVCs and complex arrhythmias from PM and outflow tract [7, 8]. Therefore, in patients with MAD and frequent symptomatic arrhythmias of PM origin, a more aggressive protocol should be employed independently of LV fibrosis in CMR. Syncope patients may be candidates for EPS, and they should be carefully monitored for progressive disease thereafter, as proposed in the Essayagh *et al*. [25] study, possibly via implantation of loop recorders [7]. However, further studies are needed to strengthen the prognostic significance of EPS in this population.

All these parameters contribute to elucidating the association between the substrate and the trigger in this arrhythmic syndrome and serve as risk markers but as discussed earlier we focus on the presence of MAD, fibrosis, PVC origin and the “positive” EPS as the strongest correlators with malignant arrhythmias and thus SCD risk predictors.

**Fig. 4.**
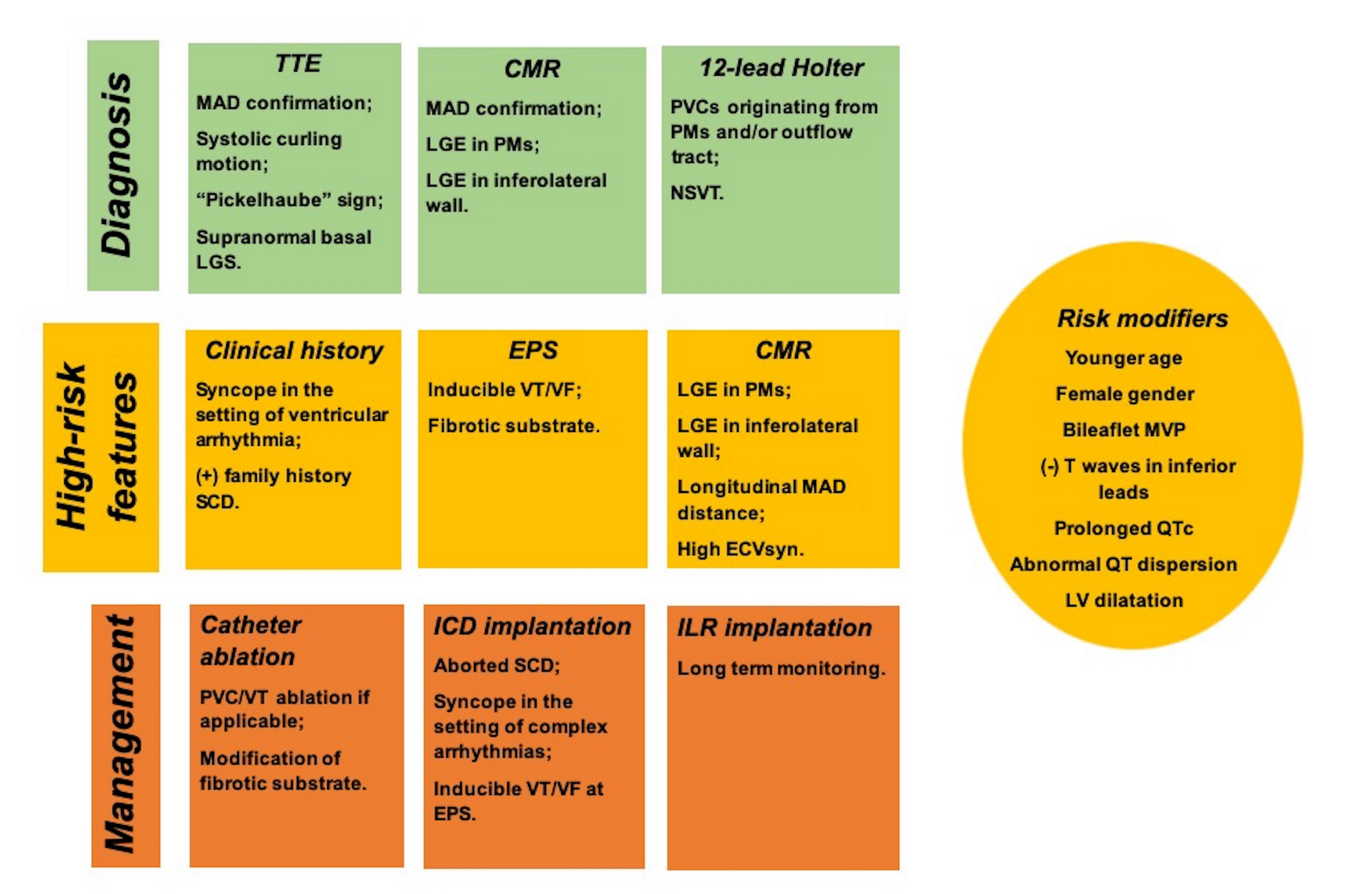
**Diagnosis, risk stratification and management of mitral annular disjunction (MAD) arrhythmic syndrome**. Abbreviations: CMR, cardiac magnetic resonance imaging; EPS, electrophysiological study; GLS, global longitudinal strain; ICD, implantable cardioverter defibrillator; ILR, implantable loop recorder; LV, left ventricle; LGE, late gadolinium enhancement; MVP, mitral valve prolapse; NSVT, non-sustained VT; PVC, premature ventricular complex; QTc, corrected QT interval; SCD, sudden cardiac death; TTE, transthoracic echocardiography; VT/VF, ventricular tachycardia/ventricular fibrillation.

The following protocol can be used for risk stratification of MAD patients. All patients with echocardiographically detected MAD accompanied by signs of mechanical stretch (high longitudinal strain values in basal inferolateral wall/“Pickelhaube” sign, inverted T waves in inferior leads) and frequent ventricular arrhythmias arising from posterior PM/outflow tract (12-lead ECG Holter monitoring and treadmill test), should be further tested with CMR to detect myocardial or PM fibrosis. In cases of symptomatic VTs or LGE/diffuse fibrosis, EPS should be performed to further map the site of ectopic activity and to reveal possible multifocality, along with programmed ventricular stimulation. Patients with “positive” EPS should be referred for implantable cardioverter defibrillator (ICD) implantation. In accordance with the recently announced risk stratification algorithm of EHRA, asymptomatic patients without complex ventricular may be further monitored with an ILR, while MVP patients with no documented arrhythmias, but other malignant phenotypic characteristics (as previously described) may be candidates for ILR, or frequent monitoring [28]. These data are depicted in Fig. 4.

## 6. Management

Management of ventricular arrhythmias in MVP patient includes anti-arrhythmic drug therapy, catheter ablation, surgical intervention and prevention of SCD with device therapy, as discussed earlier [26]. Currently, no antiarrhythmic medication has been reported to be highly effective in this population [59]. In a recent cohort, MAD strongly correlated with arrhythmic events even in patients under medication [25]. Beta-blockers are the main category used acting by reducing hypercontractility responsible for mechanical stretch to the mitral subvalvular apparatus and adjacent myocardium, but also by adjusting the catecholamine levels [26, 59].

In current practice, catheter ablation is reserved for patients with frequent symptomatic PVCs, sustained ventricular arrhythmias and VF [31]. PVC-triggered VF is the main mechanism of SCD in bileaflet MVP patients [33] and thus, identification and ablation of dominant PVC foci can be a useful strategy in this population. Ablation of idiopathic VF was first reported by Haisaguerre *et al*. [60, 61] where PVCs with short coupling interval were mapped and ablated. These PVCs where often preceded by PLPs, same as in cardiac arrest survivors with bileaflet MVP, and mainly located in PMs or fascicular tissue adjacent to PMs [33, 42].

Catheter ablation of the dominant PVC foci in MVP patient has been proved to reduce the arrhythmic burden and ICD shocks, and should be recommended, especially in patients with monomorphic PVCs [7, 33]. The PMs are the most common ablation target with varying acute success rate ranging from 60–100% both in MVP patients [47, 62, 63] and in patients with ventricular arrhythmias from PMs of other etiology, with less promising long term success rate of about 60% [46]. Other possible sites of ectopic activity amenable to radiofrequency (RF) ablation are the outflow tract and the mitral annulus [33, 47, 64]. Activation mapping of spontaneous or inducible PVCs is the main strategy [33]. The site of earliest activation located by the bipolar catheter is compared with the surface QRS of the PVC and targeted for ablation [33]. Pace mapping can also be used, taking into consideration both the morphology and latency of capture, in case of non-inducible ventricular arrhythmias [47]. An example of catheter ablation of a PVC arising from the posteromedial PM is shown in Fig. 3. RF ablation of PVCs arising from PMs poses certain limitations and repeat procedures along with late recurrence are reported in MVP patients [30, 47, 62]. In particular, RF ablation of the PMs can be extremely challenging due to difficulty in maintaining catheter stability and intracavitary position of PMs [33, 46, 65], often leading to a need for multiple procedures. Intracardiac echocardiography catheter (ICE) is commonly used to guide the procedure [65], enabling direct visualization of anatomic structures and confirming adequate ablation catheter contact at the site of earliest activation [33, 47, 62]. Cryoablation has been proposed in refractory cases for better catheter contact to the PM [43, 63, 66]. Although effective in reducing PVC burden, ablation therapy may not prevent fatal arrhythmias in MVP patients, as it is suggested in the study of Marano *et al*. [47] where 33% of patients presented with hemodynamically significant VT/VF after index ablation [47]. Interestingly, all patients who required repeat ablation had PVCs of different foci than the index procedure which is consistent with the progressive nature of the arrhythmic substrate, especially in patients with multifocal PVCs. On the other hand, EPS seems to accurately identify “high risk” patients since all of them had inducible VF in prior programmed ventricular stimulation [47]. The progressive clinical course of the substrate is also underlined in the recently published 10 year study of MAD/MVP patients where MAD correlated with long-term excess incidence of clinical arrhythmia [25].

There is a lack of evidence concerning surgical replacement or mitral valve repair and arrhythmia outcomes, with data deriving mostly from small single-center series and isolated cases [67, 68]. To date, surgical repair is indicated in severe mitral regurgitation targeting repair of the prolapsing leaflets. This is supposed to relieve, at least to some degree, the mechanical stretch to the subvalvular apparatus which acts as a trigger for arrhythmic events [68]. On the other hand, severe arrhythmias are frequently presented in patients with mild and moderate mitral regurgitation, who are not candidates for surgical intervention [6, 7]. Surgical repair cannot reverse the natural course of the disease especially when considering the involvement of fibrosis and the progressive arrhythmogenic substrate [25, 26], and thus terminate the arrhythmia circle. However, there are reports of reduction of arrhythmic burden after surgical intervention, but this may be attributed to left ventricular remodeling and load volume restoration [69]. From a technical aspect, in MAD cases, the posterior leaflet needs to be repositioned and attached to basal myocardium and further secured with an annuloplasty ring, with a reported success rate of 87% [68].

In all patients with documented VT/VF as well as in cardiac arrest survivors an ICD should be implanted as secondary prevention, in accordance with international guidelines. No specific guidance exists for primary prevention in this population. A proposed algorithm based on the available risk stratification tools for the management of patients with MAD is depicted in Fig. 4.

## 7. Conclusions

MAD is a novel “high-risk” marker among patients with or without MVP, as it is associated with malignant ventricular arrhythmias. Yet the real arrhythmic prevalence needs to be determined. Recent studies suggest the progressive course of this arrhythmic substrate, mandating careful monitoring of these patients and the need for more aggressive risk stratification protocols. The longitudinal MAD distance as well as the detection of fibrotic arrhythmogenic substrate with CMR are of prognostic significance for arrhythmias. EPS appears to have a good correlation with identification of “high-risk” patients suitable for ICD implantation, and in that direction more prospective studies are needed to further establish its role in the primary prevention strategy.

## References

[1] Hutchins GM, Moore GW, Skoog DK (1986). The association of floppy mitral valve with disjunction of the Mitral annulus fibrosus. *The New England Journal of Medicine*.

[2] Eriksson MJ, Bitkover CY, Omran AS, David TE, Ivanov J, Ali MJ (2005). Mitral Annular Disjunction in Advanced Myxomatous Mitral Valve Disease: Echocardiographic Detection and Surgical Correction. *Journal of the American Society of Echocardiography*.

[3] Basso C, Perazzolo Marra M, Rizzo S, De Lazzari M, Giorgi B, Cipriani A (2015). Arrhythmic Mitral Valve Prolapse and Sudden Cardiac Death. *Circulation*.

[4] Perazzolo Marra M, Basso C, De Lazzari M, Rizzo S, Cipriani A, Giorgi B (2016). Morphofunctional Abnormalities of Mitral Annulus and Arrhythmic Mitral Valve Prolapse. *Circulation: Cardiovascular Imaging*.

[5] Carmo P, Andrade MJ, Aguiar C, Rodrigues R, Gouveia R, Silva JA (2010). Mitral annular disjunction in myxomatous mitral valve disease: a relevant abnormality recognizable by transthoracic echocardiography. *Cardiovascular Ultrasound*.

[6] Dejgaard LA, Skjølsvik ET, Lie ØH, Ribe M, Stokke MK, Hegbom F (2018). The Mitral Annulus Disjunction Arrhythmic Syndrome. *Journal of the American College of Cardiology*.

[7] Sriram CS, Syed FF, Ferguson ME, Johnson JN, Enriquez-Sarano M, Cetta F (2013). Malignant Bileaflet Mitral Valve Prolapse Syndrome in Patients with otherwise Idiopathic out-of-Hospital Cardiac Arrest. *Journal of the American College of Cardiology*.

[8] Hourdain J, Clavel MA, Deharo J, Asirvatham S, Avierinos JF, Habib G (2018). Common Phenotype in Patients with Mitral Valve Prolapse who Experienced Sudden Cardiac Death. *Circulation*.

[9] Delling FN, Rong J, Larson MG, Lehman B, Fuller D, Osypiuk E (2016). Evolution of Mitral Valve Prolapse. *Circulation*.

[10] Freed LA, Levy D, Levine RA, Larson MG, Evans JC, Fuller DL (1999). Prevalence and Clinical Outcome of Mitral-Valve Prolapse. *New England Journal of Medicine*.

[11] Anyanwu AC, Adams DH (2007). Etiologic classifification of degenerative mitral valve disease: Barlow’s disease and fifibroelastic defificiency. *Seminars in Thoracic and Cardiovascular Surgery*.

[12] Van Wijngaarden AL, Kruithof BPT, Vinella T, Barge-Schaapveld DQCM, Ajmone Marsan N (2021). Characterization of degenerative mitral valve disease: Differences between fifibroelasti defificiency and Barlow’s disease. *Journal of Cardiovascular Development and Disease*.

[13] Mantegazza V, Volpato V, Gripari P, Ghulam Ali S, Fusini L, Italiano G (2021). Multimodality imaging assessment of mitral annular disjunction in mitral valve prolapse. *Heart*.

[14] Konda T, Tani T, Suganuma N, Nakamura H, Sumida T, Fujii Y (2017). The analysis of mitral annular disjunction detected by echocardiography and comparison with previously reported pathological data. *Journal of Echocardiography*.

[15] Romero Daza A, Chokshi A, Pardo P, Maneiro N, Guijarro Contreras A, Larrañaga-Moreira JM (2021). Mitral valve prolapse morphofunctional features by cardiovascular magnetic resonance: more than just a valvular disease. *Journal of Cardiovascular Magnetic Resonance*.

[16] Punjabi PP, Rana BS (2021). Mitral annular disjunction: is MAD ‘normal’. *European Heart Journal - Cardiovascular Imaging*.

[17] Faletra FF, Leo LA, Paiocchi VL, Schlossbauer SA, Pavon AG, Ho SY (2022). Morphology of Mitral Annular Disjunction in Mitral Valve Prolapse. *Journal of the American Society of Echocardiography*.

[18] Angelini A, Ho SY, Anderson RH, Becker AE, Davies MJ (1988). Disjunction of the mitral annulus in floppy mitral valve. *The New England Journal of Medicine*.

[19] Putnam AJ, Kebed K, Mor-Avi V, Rashedi N, Sun D, Patel B (2020). Prevalence of mitral annular disjunction in patients with mitral valve prolapse and severe regurgitation. *The International Journal of Cardiovascular Imaging*.

[20] Zhou N, Zhao Q, Zeng X, Zheng D, Yue J, Zhang K (2021). Association of Mitral Annular Disjunction with Premature Cardiac Mortality in a Large Series of Autopsies. *Journal of the American College of Cardiology*.

[21] Bennett S, Thamman R, Griffiths T, Oxley C, Khan JN, Phan T (2019). Mitral annular disjunction: a systematic review of the literature. *Echocardiography*.

[22] Basso C, Calabrese F, Corrado D, Thiene G (2001). Postmortem diagnosis in sudden cardiac death victims: macroscopic, microscopic and molecular findings. *Cardiovascular Research*.

[23] Nalliah CJ, Mahajan R, Elliott AD, Haqqani H, Lau DH, Vohra JK (2019). Mitral valve prolapse and sudden cardiac death: a systematic review and meta-analysis. *Heart*.

[24] Miller MA, Dukkipati SR, Turagam M, Liao SL, Adams DH, Reddy VY (2018). Arrhythmic Mitral Valve Prolapse. *Journal of the American College of Cardiology*.

[25] Essayagh B, Sabbag A, Antoine C, Benfari G, Batista R, Yang L (2021). The Mitral Annular Disjunction of Mitral Valve Prolapse. *JACC: Cardiovascular Imaging*.

[26] Basso C, Iliceto S, Thiene G, Perazzolo Marra M (2019). Mitral Valve Prolapse, Ventricular Arrhythmias, and Sudden Death. *Circulation*.

[27] Wibawa K, Ivan I, Jessica G, Ridjab D (2021). The Outcome of Ventricular Arrhythmias Associated with Mitral Valve Prolapse after Catheter Ablation: a Systematic Review and Meta-Analysis. *Cureus*.

[28] Sabbag A EHRA expert consensus statement on arrhythmic mitral valve prolapse and mitral annular disjunction complex. https://esc365.escardio.org/event/376.

[29] Appignani M, Khanji MY, Arbustini E, Stuppia L, Ceriello L, Girolamo ED (2021). Is Occult Genetic Substrate the Missing Link between Arrhythmic Mitral Annular Disjunction Syndrome and Sudden Cardiac Death. *Canadian Journal of Cardiology*.

[30] Essayagh B, Sabbag A, Antoine C, Benfari G, Yang L, Maalouf J (2020). Presentation and Outcome of Arrhythmic Mitral Valve Prolapse. *Journal of the American College of Cardiology*.

[31] Chakrabarti AK, Bogun F, Liang JJ (2022). Arrhythmic Mitral Valve Prolapse and Mitral Annular Disjunction: Clinical Features, Pathophysiology, Risk Stratification, and Management. *Journal of Cardiovascular Development and Disease*.

[32] Guenancia C, Pace N, Hossu G, Selton-Suty C, Mandry D, Beaumont M (2022). Prevalence and Determinants of PVCs Originating from the Mitral Apparatus in Patients with MVP. *JACC: Clinical Electrophysiology*.

[33] Syed FF, Ackerman MJ, McLeod CJ, Kapa S, Mulpuru SK, Sriram CS (2016). Sites of Successful Ventricular Fibrillation Ablation in Bileaflet Mitral Valve Prolapse Syndrome. *Circulation: Arrhythmia and Electrophysiology*.

[34] Lee AP, Jin C, Fan Y, Wong RHL, Underwood MJ, Wan S (2017). Functional Implication of Mitral Annular Disjunction in Mitral Valve Prolapse. *JACC: Cardiovascular Imaging*.

[35] Grewal J, Suri R, Mankad S, Tanaka A, Mahoney DW, Schaff HV (2010). Mitral Annular Dynamics in Myxomatous Valve Disease. *Circulation*.

[36] Wunderlich NC, Ho SY, Flint N, Siegel RJ (2021). Myxomatous Mitral Valve Disease with Mitral Valve Prolapse and Mitral Annular Disjunction: Clinical and Functional Significance of the Coincidence. *Journal of Cardiovascular Development and Disease*.

[37] Salgo IS, Gorman JH, Gorman RC, Jackson BM, Bowen FW, Plappert T (2002). Effect of annular shape on leaflflet curvature in reducing mitral leaflet stress. *Circulation*.

[38] Silbiger JJ (2012). Anatomy, mechanics, and pathophysiology of the mitral annulus. *American Heart Journal*.

[39] Muthukumar L, Rahman F, Jan MF, Shaikh A, Kalvin L, Dhala A (2017). The Pickelhaube Sign. *JACC: Cardiovascular Imaging*.

[40] Constant Dit Beaufils AL, Huttin O, Jobbe-Duval A, Cueff C, Piriou N, Senage T (2021). Replacement myocardial fibrosis in patients with mitral valve prolapse. Relation to mitral regurgitation, ventricular remodeling and arrhythmia. *Archives of Cardiovascular Diseases Supplements*.

[41] Boyden PA, Hirose M, Dun W (2010). Cardiac Purkinje cells. *Heart Rhythm*.

[42] Santoro F, Biase LD, Hranitzky P, Sanchez JE, Santangeli P, Perini AP (2014). Ventricular Fibrillation Triggered by PVCs from Papillary Muscles: Clinical Features and Ablation. *Journal of Cardiovascular Electrophysiology*.

[43] Franz MR, Cima R, Wang D, Profitt D, Kurz R (1992). Electrophysiological effects of myocardial stretch and mechanical determinants of stretch-activated arrhythmias. *Circulation*.

[44] Sedmera D, Gourdie RG (2014). Why do we have Purkinje Fibers Deep in our Heart. *Physiological Research*.

[45] Choquet C, Boulgakoff L, Kelly RG, Miquero L (2021). New Insights into the Development and Morphogenesis of the Cardiac Purkinje Fiber Network: Linking Architecture and Function. *Journal of Cardiovascular Development and Disease*.

[46] Raja DC, Rangaswamy VV, Abhilash SP, King K, Pathak RK (2020). Electrophysiological Substrates in Papillary Muscle Arrhythmias – Implications for Catheter Ablation. *European Journal of Arrhythmia & Electrophysiology*.

[47] Marano PJ, Lim LJ, Sanchez JM, Alvi R, Nah G, Badhwar N (2021). Long-term outcomes of ablation for ventricular arrhythmias in mitral valve prolapse. *Journal of Interventional Cardiac Electrophysiology*.

[48] Pavon AG, Arangalage D, Pascale P, Hugelshofer S, Rutz T, Porretta AP (2021). Myocardial extracellular volume by T1 mapping: a new marker of arrhythmia in mitral valve prolapse. *Journal of Cardiovascular Magnetic Resonance*.

[49] Han Y, Peters DC, Salton CJ, Bzymek D, Nezafat R, Goddu B (2008). Cardiovascular Magnetic Resonance Characterization of Mitral Valve Prolapse. *JACC: Cardiovascular Imaging*.

[50] Bekheit SG, Ali AA, Deglin SM, Jain AC (1982). Analysis of QT Interval in Patients with Idiopathic Mitral Valve Prolapse. *Chest*.

[51] Kulan K, Komsuoglu B, Tuncer C, Kulan C (1996). Signifificance of QT dispersion on ventricular arrhythmias in mitral valve prolapse. *International Journal of Cardiology*.

[52] Gilbert BW, Schatz RA, VonRamm OT, Behar VS, Kisslo JA (1976). Mitral valve prolapse. Two-dimensional echocardiographic and angiographic correlation. *Circulation*.

[53] Muthukumar L, Jahangir A, Jan MF, Galazka P, Umland M, Schweitzer MR (2020). Left Ventricular Global and Regional Deformation in Arrhythmic Myxomatous Bileaflet Mitral Valve Prolapse Syndrome. *JACC: Cardiovascular Imaging*.

[54] Muthukumar L, Jahangir A, Jan MF, Perez Moreno AC, Khandheria BK, Tajik AJ (2020). Association between Malignant Mitral Valve Prolapse and Sudden Cardiac Death. *JAMA Cardiology*.

[55] Wang TKM, Kwon DH, Abou-Hassan O, Chetrit M, Harb SC, Patel D (2021). Strain evaluation for mitral annular disjunction by echocardiography and magnetic resonance imaging: a case-control study. *International Journal of Cardiology*.

[56] Jaworski K, Firek B, Syska P, Lewandowski M, Śpiewak M, Dąbrowski R (2022). Malignant arrhythmia associated with mitral annular disjunction: Myocardial work as a potential tool in the search for a substrate. *Kardiologia Polska*.

[57] Kawakami H, Nerlekar N, Haugaa KH, Edvardsen T, Marwick TH (2020). Prediction of Ventricular Arrhythmias with Left Ventricular Mechanical Dispersion. *JACC: Cardiovascular Imaging*.

[58] Ermakov S, Gulhar R, Lim L, Bibby D, Fang Q, Nah G (2019). Left ventricular mechanical dispersion predicts arrhythmic risk in mitral valve prolapse. *Heart*.

[59] Hong-TaoYuan, Yang M, Zhong L, Lee Y, Vaidya VR, Asirvatham SJ (2016). Ventricular premature contraction associated with mitral valve prolapse. *International Journal of Cardiology*.

[60] Haïssaguerre M, Shah DC, Jaïs P, Shoda M, Kautzner J, Arentz T (2002). Role of Purkinje conducting system in triggering of idiopathic ventricular fibrillation. *The Lancet*.

[61] Haïssaguerre M, Shoda M, Jaïs P, Nogami A, Shah DC, Kautzner J (2002). Mapping and Ablation of Idiopathic Ventricular Fibrillation. *Circulation*.

[62] Enriquez A, Shirai Y, Huang J, Liang J, Briceño D, Hayashi T (2019). Papillary muscle ventricular arrhythmias in patients with arrhythmic mitral valve prolapse: Electrophysiologic substrate and catheter ablation outcomes. *Journal of Cardiovascular Electrophysiology*.

[63] Lee A, Hamilton‐Craig C, Denman R, Haqqani HM (2018). Catheter ablation of papillary muscle arrhythmias: Implications of mitral valve prolapse and systolic dysfunction. *Pacing and Clinical Electrophysiology*.

[64] Bumgarner JM, Patel D, Kumar A, Clevenger JR, Trulock KM, Popović Z (2019). Management and outcomes in mitral valve prolapse with ventricular arrhythmias undergoing ablation and/or implantation of ICDs. *Pacing and Clinical Electrophysiology*.

[65] Lin AN, Shirai Y, Liang JJ, Chen S, Kochar A, Hyman MC (2020). Strategies for Catheter Ablation of Left Ventricular Papillary Muscle Arrhythmias. *JACC: Clinical Electrophysiology*.

[66] Rivera S, Ricapito MDLP, Tomas L, Parodi J, Bardera Molina G, Banega R (2016). Results of Cryoenergy and Radiofrequency-Based Catheter Ablation for Treating Ventricular Arrhythmias Arising from the Papillary Muscles of the Left Ventricle, Guided by Intracardiac Echocardiography and Image Integration. *Circulation: Arrhythmia and Electrophysiology*.

[67] Naksuk N, Syed FF, Krittanawong C, Anderson MJ, Ebrille E, DeSimone CV (2016). The effect of mitral valve surgery on ventricular arrhythmia in patients with bileaflet mitral valve prolapse. *Indian Pacing and Electrophysiology Journal*.

[68] Newcomb AE, David TE, Lad VS, Bobiarski J, Armstrong S, Maganti M (2008). Mitral valve repair for advanced myxomatous degeneration with posterior displacement of the mitral annulus. *The Journal of Thoracic and Cardiovascular Surgery*.

[69] Kay JH, Krohn BG, Zubiate P, Hoffman RL (1979). Surgical correction of severe mitral prolapse without mitral insufficiency but with pronounced cardiac arrhythmias. *The Journal of Thoracic and Cardiovascular Surgery*.

